# Impact of Hydroxyurea Therapy in Reducing Pain Crises, Hospital Admissions, and Length of Stay Among Sickle Cell Patients in the Eastern Region of Saudi Arabia

**DOI:** 10.7759/cureus.31527

**Published:** 2022-11-15

**Authors:** Hassan Albohassan, Muhammed Ammen, Ali A Alomran, Hussain Bu Shehab, Hussain Al Sakkak, Afnan Al Bohassan

**Affiliations:** 1 Pediatrics, Almoosa Specialist Hospital, Al-Ahsa, SAU; 2 Pediatric Hematology/Oncology, Almoosa Specialist Hospital, Al-Ahsa, SAU; 3 Urology, Prince Saud Bin Jalawi Hospital, Al-Ahsa, SAU; 4 Medicine and Surgery, University of Pécs, Pècs, HUN; 5 Medicine and Surgery, King Faisal University, Al-Ahsa, SAU

**Keywords:** length of hospitalization, vaso-occlusive crisis, hydroxyurea, sickle cell complications, sickle cell disease (scd)

## Abstract

Background

In Saudi Arabia, sickle cell disease (SCD) is a major public health issue, especially in the eastern region. Sickle cell patients have major health-related issues, resulting in a poor quality of life and increased morbidity. Abnormal hemoglobin production in SCD causes various complications, such as vaso-occlusive crises, hemolytic episodes, and acute chest syndrome. These disease manifestations increase the need for hospital admission and long-term care. Most therapies for SCD are supportive and include episodic red blood cell transfusions, narcotics, antibiotics, and intravenous fluids. Hydroxyurea is a disease-modifying therapy. This study aimed to assess the effectiveness of hydroxyurea therapy on reducing pain crises, hospital admissions, and length of stay for SCD patients and discern reasons that would prevent SCD patients from using hydroxyurea as a treatment option.

Methodology

We conducted a descriptive cross-sectional study on SCD patients from the eastern Saudi Arabian province. The study included 202 SCD patients from hematology clinics and medical wards. We used a validated questionnaire tested for reliability after a pilot study of 15 randomly selected patients. We surveyed participants on demographic data, use of hydroxyurea, compliance with the regimen, hospitalization rates, durations, and complications. The study used IBM SPSS Statistics for Windows, Version 22.0. (IBM Corp., Armonk, NY) to analyze the data. All statistical analysis was done using two-tailed tests. P-values < 0.05 were considered statistically significant.

Results

The study included 202 participants who agreed to participate and completed the study questionnaire. The respondents comprised 150 SCD patients (74.3%) and 52 caregivers of SCD patients (25.7%). Patient ages ranged from one year to older than 36 years (mean age: 26.8 ± 12.3 years). The most common reason for not using hydroxyurea was that it was never offered to patients as a treatment option (40.9% of respondents), followed by respondents who had never heard of it (34.4%), think that they do not need it (24.7%), and fears of long-term consequences (23.7%). More hydroxyurea users (35.1%) suffered no acute painful crises during the last year than nonusers (32%), and more nonusers suffered more than four crises (20%) than hydroxyurea users (9.1%; p=.046). As for hospitalization due to SCD-related complications, 66.2% of hydroxyurea users were never hospitalized, while 51.2% of nonusers were never hospitalized. While 19.5% of hydroxyurea users were hospitalized one to two times, 28.8% of nonusers were hospitalized one to two times (p=.049).

Conclusions

The study revealed that hydroxyurea is effective in reducing painful vaso-occlusive crises and the number of hospital admissions. The prevalence of hydroxyurea use among SCD patients in the eastern province of Saudi Arabia remains low. Therefore, education campaigns and programs to increase awareness among health care providers regarding the benefits of hydroxyurea use are warranted in our region to help improve patient outcomes.

## Introduction

Genetic hemoglobinopathies are becoming a global health burden, with an estimated more than 300,000 children born each year with severe hereditary hemoglobin disorders [1]. One of these is sickle cell disease (SCD), an autosomal recessive disease characterized by the production of abnormal hemoglobin (Hb) S [2]. Biswas predicted that the global number of newborn babies with SCD will increase by one-third in 2050 [3].

SCD is a significant public health issue in Saudi Arabia, especially in the eastern region, where it has the highest prevalence [2]. SCD patients have significant health issues that result in a poor quality of life and morbidity. The abnormal Hb production in SCD gives red blood cells (RBCs) a sickle-like shape in a state of hypoxia, resulting in a broad spectrum of complications such as vaso-occlusive crises, hemolytic episodes, stroke, acute chest syndrome, and vulnerability to various infections [4].

In 1998, the Food and Drug Administration (FDA) approved hydroxyurea as a therapeutic agent for the treatment of SCD [5]. Before hydroxyurea's approval, most SCD therapies were supportive (e.g., episodic red blood cell transfusions, narcotics, antibiotics, and intravenous fluids). Hydroxyurea was the first FDA-approved therapeutic drug treatment for SCD, but several other novel therapeutic agents have emerged, such as Endari, which was approved by the FDA in 2017 [5,6]. Hydroxyurea's potential adverse effects include anorexia, nausea, vomiting, and infertility. Other serious adverse effects require periodic monitoring, such as increased liver enzymes and bone marrow suppression [7]. However, hydroxyurea's mechanism of action offers many benefits for SCD treatment; most importantly is the increased HbF production. Other mechanisms that may play a role in SCD treatment are increasing nitric oxide, enhancing RBC rheology, and reducing white blood cell counts [8].

Hydroxyurea's efficacy in reducing the frequency of painful crises and hospitalization has been studied several times [9-12], but never in eastern Saudi Arabia. Therefore, we conducted this study to assess the effectiveness of hydroxyurea therapy in reducing pain crises, hospital admissions, and length of stay for SCD patients in the eastern region of Saudi Arabia and discern reasons that would prevent SCD patients from using hydroxyurea as a treatment option.

## Materials and methods

We conducted a descriptive cross-sectional study on SCD patients from the eastern Saudi Arabian province. Study participants were recruited from hematology clinics and sickle cell society groups and completed a validated questionnaire that was tested for reliability after a pilot study of 15 randomly selected patients. Participants were included in the study if they had a confirmed diagnosis of SCD or were the caretaker of patients with confirmed SCD. SCD patients were included regardless of hydroxyurea use. The study excluded infants younger than one year.

Data were collected through a validated anonymous questionnaire. The questionnaire was tested for content, readability, and comprehension, and it was designed in a pilot study on 15 randomly selected patients from the survey's population pool. After piloting and analyzing the respondents' feedback, the questionnaire was finalized after ambiguous, and unsuitable questions were improved or removed based on the pilot study results. We collected SCD patient demographic data, use of hydroxyurea, compliance with the regimen, hospitalization rates, durations, and complications.

Statistical analysis

Data were coded and analyzed to statistical software IBM SPSS Statistics for Windows, Version 22.0. (IBM Corp., Armonk, NY). All statistical analysis was done using two-tailed tests. A p-value less than 0.05 was considered to be statistically significant. Descriptive analysis based on the frequency and percent distribution was done for all variables, including participants' demographic data, hydroxyurea use data, and SCD status, including hospitalization and complications. Cross tabulation was used to test the number of pain crises, hospital admissions, and length of stay in SCD patients by hydroxyurea use status (users vs. nonusers). Pearson chi-square test was used to test for relationship significance.

## Results

**Figure 1:**
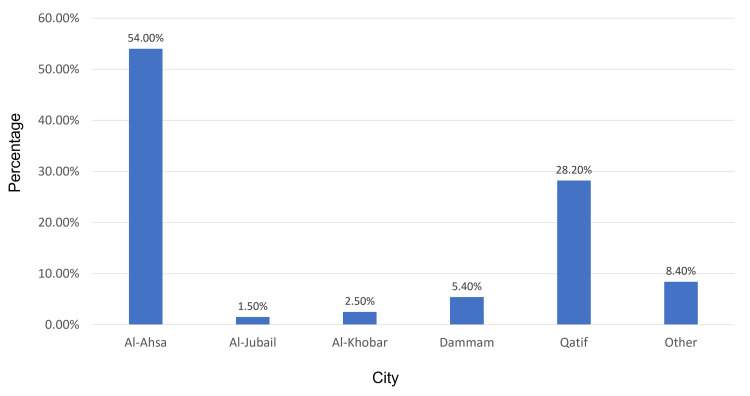
Study population distribution among cities in eastern Saudi Arabia

**Figure 2:**
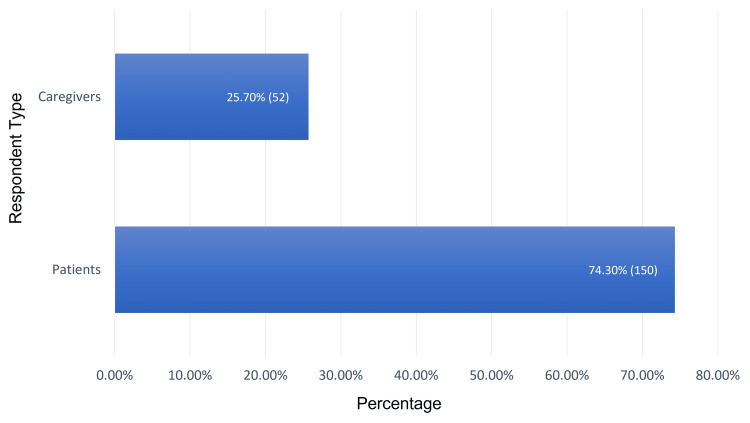
Study respondent distribution of caregivers and patients

**Figure 3:**
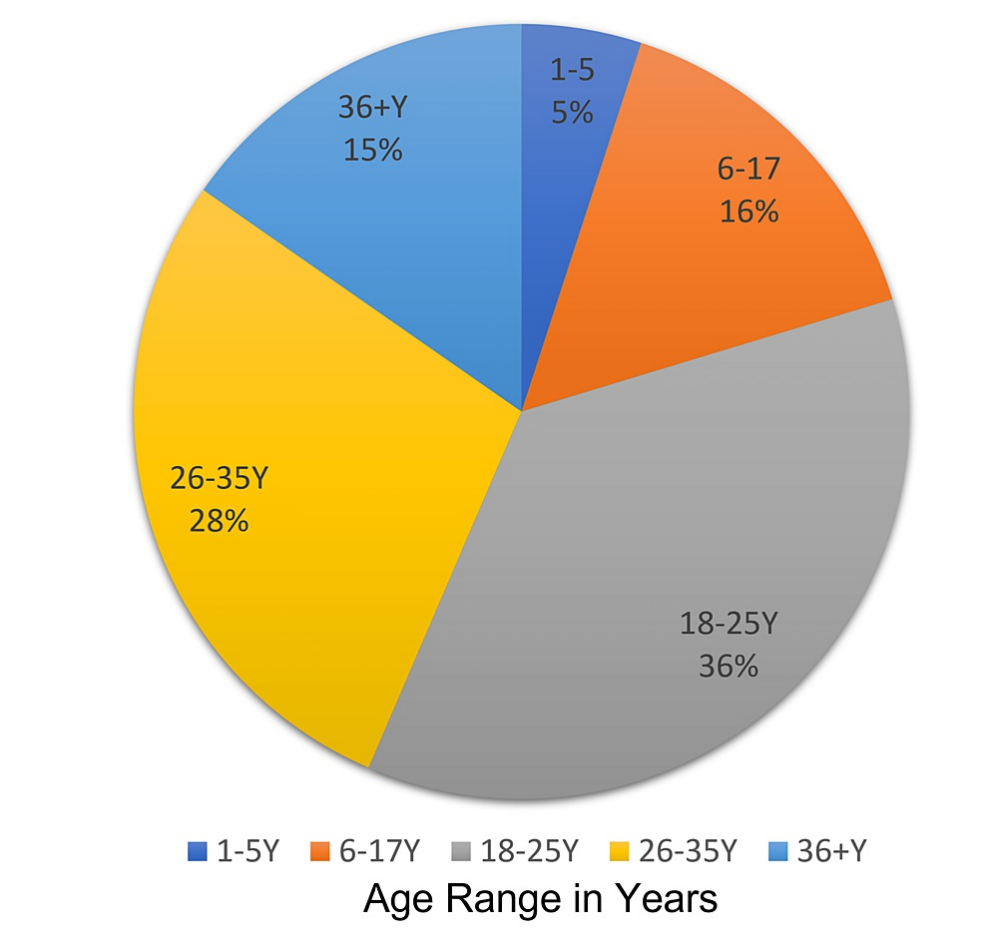
Respondent age distribution

The study included 202 participants who agreed to participate and completed the study questionnaire. Most respondents were from Al-Ahasa city (54%; n=109; Figure 1). Our study’s respondents included 150 SCD patients (74.3%) and 52 caregivers of SCD patients (25.7%; Figure 2). Patients' ages ranged from one year to more than 36 years, with a mean age of 26.8 ± 12.3 years (Figure 3).

Table 1 shows hydroxyurea use data among patients with SCD in our study. Seventy-seven patients (38.1%) currently use hydroxyurea for SCD pain crises, 32 (15.8%) stopped using hydroxyurea, and 93 (46%) have never used it. Most hydroxyurea users (n=66; 85.7%) had used it for at least one year. Among patients who have not used hydroxyurea, the number one reason for lack of use was that it was never offered to patients as a treatment option (40.9%), followed by lack of awareness of it (34.4%), thinking they do not need it (24.7%), and being afraid of long-term consequences (23.7%). Among patients who stopped using hydroxyurea, 34.4% thought they did not need it, 28.1% could not tolerate it, 28.1% were concerned about long-term consequences, and 12.5% were unavailable for follow-up. Some respondents felt it was only helpful for the first year and had headaches, nausea, or fatigue. Fifty-six patients (72.7%) strongly agreed with the statement that they take hydroxyurea regularly as per their doctor's instructions, while 16 (20.8%) reported agreement only.

**Table 1:** Hydroxyurea use-related data among patients with SCD in Eastern Saudi Arabia SCD: Sickle cell disease

Hydroxyurea use	N	%
Do you use Hydroxyurea as treatment for sickle cell disease?		
Never use Hydroxyurea	93	46.0%
Stopped using Hydroxyurea	32	15.8%
Currently using Hydroxyurea	77	38.1%
Duration of using Hydroxyurea for sickle cell disease treatment (n=77)		
Less than 1 year	11	14.3%
More than one year	66	85.7%
Reason of never using Hydroxyurea (n=93)		
Never heard about it	32	34.4%
Never offered to me as treatment option	38	40.9%
My doctor think, I don’t need it	18	19.4%
I think that I don’t need it	23	24.7%
I am afraid of long-term consequences	22	23.7%
Reason for stopping Hydroxyurea (n=32)		
No available follow up	4	12.5%
I cannot tolerate the side effect	9	28.1%
My doctor think, I don’t need it	2	6.3%
I think that I don’t need it	11	34.4%
I am afraid of long-term consequences	9	28.1%
Others	6	18.8%
I often take Hydroxyurea medication regularly as per my doctor's instructions		
Strongly agree	56	72.7%
To some extent agree	5	6.5%
Agree	16	20.8%

Table 2 shows SCD status as reported by study participants. Sixty-seven patients (33.2%) reported that they had not suffered any acute painful crises during the last year, 68 (33.7%) had acute painful crises once or twice, and 32 (15.8%) had more than four crises in the last year. Also, 115 patients (56.9%) were never hospitalized during the year before the study, 51 (25.2%) were hospitalized one to two times, and 15 (7.4%) were hospitalized more than four times. Thirty-seven patients (42.5%) were hospitalized for SCD issues for three to five days, 23 (26.4%) were hospitalized for six to 10 days, and 18 (20.7%) were hospitalized for more than 10 days. Acute painful crisis was the most common SCD complication reported (92%), followed by severely dropped Hb (39.1%), acute chest syndrome (25.3%), and avascular necrosis of the hip (20.7%).

**Table 2:** SCD status as reported by study participants in Eastern Saudi Arabia SCD: Sickle cell disease

SCD status	N	%
Last year, how many times have you suffered from acute painful crises?		
I haven’t suffered any acute painful crises	67	33.2%
1-2 times	68	33.7%
3-4 times	35	17.3%
More than 4 times	32	15.8%
Last year, how many times I had to be hospitalized due to sickle cell-related complications		
Never hospitalized	115	56.9%
1-2 times	51	25.2%
3-4 times	21	10.4%
More than 4 times	15	7.4%
Last year, what was the longest period of hospitalization due to sickle cell crisis? (n=87)		
Two days and less	9	10.3%
3-5 days	37	42.5%
6-10 days	23	26.4%
More than 10 days	18	20.7%
What were the sickle cell disease complications that you had to be admitted for? (n=87)		
Acute painful crises	80	92.0%
Severely dropped Hemoglobin	34	39.1%
Acute Chest Syndrome	22	25.3%
Splenic sequestration	12	13.8%
Avascular necrosis of the hip	18	20.7%
Others	9	10.3%
Cholecystitis or gallstones	4	4.6%
Hepatic crisis	1	1.1%

Table 3 reveals the distribution of SCD status by hydroxyurea users. A more significant proportion of hydroxyurea users (35.1%) had not suffered from an acute painful crisis last year than nonusers (32%). Suffering pain more than four times per day was significantly more prevalent among nonusers (20% of nonusers vs. 9.1% of users; p=.046). A larger majority (66.2%) of hydroxyurea users were never hospitalized than nonusers (51.2%). Significantly more nonusers were hospitalized once or twice (28.8%) than users (19.5%; p=.049). Regarding hospitalization duration, 38.5% of hydroxyurea users were hospitalized for six to 10 days compared to 21.3% nonusers. More nonusers (24.6%) were hospitalized for more than 10 days than users (11.5%), but the difference was not statistically significant (p=.286).

**Table 3:** Distribution of SCD status by hydroxyurea use SCD: Sickle cell disease

SCD status	Hydroxyurea using as treatment for SCD				P-value
	Non-Hydroxyurea users		Hydroxyurea users		
	No	%	No	%	
Last year, how many times have you suffered from acute painful crises?					.046*
I haven’t suffered any acute painful crises	40	32.0%	27	35.1%	
1-2 times	43	34.4%	25	32.5%	
3-4 times	17	13.6%	18	23.4%	
More than 4 times	25	20.0%	7	9.1%	
Last year, how many times I had to be hospitalized due to sickle cell-related complications					.049*
Never hospitalized	64	51.2%	51	66.2%	
1-2 times	36	28.8%	15	19.5%	
3-4 times	15	12.0%	6	7.8%	
More than 4 times	10	8.0%	5	6.5%	
Last year, what was the longest period of hospitalization due to sickle cell crisis?					.286
Two days and less	7	11.5%	2	7.7%	
3-5 days	26	42.6%	11	42.3%	
6-10 days	13	21.3%	10	38.5%	
More than 10 days	15	24.6%	3	11.5%	

## Discussion

The current study was conducted to assess the impact of hydroxyurea therapy in reducing pain crises, hospital admissions, and length of stay among SCD patients. Pain is prevalent among SCD patients and is the main reported concern [13,14]. Pain in SCD ranges from mild to severe intensity and is typically sharp or pulsing but can also be stabbing, deep, achy, lacerating, or shooting in some cases [15,16]. Tolerable pain (i.e., mild or moderate pain) is frequently handled at home with oral or topical analgesics, besides some nonpharmacological procedures [14,17]. Severe pain is regularly managed in the emergency department and/or hospital inpatient setting with parenteral opioids. A few randomized controlled trials were conducted to compare the effect of drug treatment on the length of hospital stay for painful crises among adult patients with SCD [18,19]. Anecdotes and reports from different studies indicate that the duration of hospitalization for an uncomplicated painful crisis in adult patients varies from six to 10 days [20,21].

Our study found that slightly more than half of the patients never used hydroxyurea in treating SCD, while only one-third currently use the drug, but a lower percentage previously had used the drug but stopped. A study in Oman found that most patients who use hydroxyurea regularly adhered to their physicians' instructions (82.5%) based on self-reported adherence. Of 298 patients studied, 128 (43.0%) reported using hydroxyurea at some point [22]. We found that 85.7% of patients use the drug for more than one year, and 72.7% strongly agreed that they follow their doctors' instructions regularly. Among those who never used the drug, more than three-quarters had either never heard of the drug or the drug was never offered as a treatment modality, which may be a lacking from the healthcare side or noncompliance from patients for follow-up. A systemic review of the adoption of hydroxyurea revealed that only 44% of physicians prescribe hydroxyurea routinely for SCD treatment [23]. Among hydroxyurea users, one-third had a negative perception regarding their need for the drug; this may be due to a lack of knowledge about the drug's efficacy or because the patient may not experience pain crises frequently. Others were afraid of the long-term consequences of the drug, including fears around conception. Intolerance to the drug's adverse effects was the third most reported cause (nearly one-fifth of the patients).

Regarding SCD status, the current study showed that two-thirds of the patients experienced acute painful crises during the last year before the study period. One-third of those patients had one to two pain crises, while a smaller proportion had pain more than four times in the last year. An autonomic nervous system (ANS) deterioration is significantly associated with SCD crisis and complications [24]. Inamo et al. reported decreased ANS activity compared to healthy controls, but ANS activity reduction varied greatly from one patient to another [25]. Pearson et al. and Romero Mestre et al. indicated a potential link between autonomic reactivity and the clinical severity of SCA [26,27]. Although it was documented that ANS activity could be implicated in the pathophysiology of SCA, the processes by which ANS imbalance may manipulate the clinical severity of SCA are still poorly understood [27,28]. Considering hospitalization, we found that more than half of the patients were never hospitalized, and of those who were, fewer than half were admitted for three to five days. Acute pain crisis was the most reported cause of hospitalization (among more than 90%) of the patients, followed by severely dropped Hb and acute chest syndrome.

Considering the role of hydroxyurea in treating SCD, we found a significant relationship between using hydroxyurea and a reduction in the frequency of acute pain crises (but not total prevention of pain crises). Also, a significantly higher hospitalization rate was reported among hydroxyurea nonusers than among current users. On the other hand, using hydroxyurea insignificantly affected the duration of hospitalization. These findings are consistent with those reported in the literature. Charache et al. reported that hydroxyurea decreased the frequency of painful crises that needed hospitalization. However, it did not address the duration of the painful crises or the amounts of opioids used to control crisis pain during hospitalization; the study did not differentiate between responders to hydroxyurea and nonresponders [10]. Ballas et al. also reported that responders to hydroxyurea used analgesics less often than nonresponders [12]. During hospitalization, 96% were treated with parenteral opioids (meperidine was most frequently used); oxycodone was the most common oral medication. The average length of stay for responders to hydroxyurea was approximately two days fewer than for other groups, and their cumulative time during the trial was significantly less than nonresponders or placebo groups [12]. Ofakunrin et al. conducted a quasi-experimental study to evaluate the effectiveness and safety of hydroxyurea among SCD patients and reported that 50% of patients had more than two episodes of painful crises before hydroxyurea use, and only 2.7% had more than two episodes of painful crises after hydroxyurea use. Likewise, 11.1% had acute chest syndrome prior to hydroxyurea use, and none had acute chest syndrome after hydroxyurea use. The risk of hospital stay for longer than seven days was 0.08 times the risk at the baseline [29]. Another study also confirmed the effect of hydroxyurea on SCD pain crisis and complications [30].

Our study had several important limitations. First, our participants were not recruited from all other Saudi Arabia provinces; rather, we focused on the Eastern province and therefore, our results are not generalizable to the rest of the country. Additionally, non-response bias may affect our questionnaire-based study, given that we required a detailed history, and meeting this requirement might have been challenging for participants.

## Conclusions

We conducted this study to assess the effectiveness of hydroxyurea therapy on reducing pain crises, hospital admissions, and length of stay for SCD patients and discern reasons that would prevent SCD patients from using hydroxyurea as a treatment option in the eastern province of Saudi Arabia where the prevalence of SCD is high. Hydroxyurea is a therapeutic option to reduce clinical sequela and improve the quality of life of SCD patients. We found a significant decline in the incidence of painful crises and duration of hospitalization in hydroxyurea users compared to nonusers. Lack of patient awareness of hydroxyurea was the most common reason for not using it. This suggests a lack of patient education and accessibility when physicians discuss patient treatment options in this region. To improve the care of SCD patients in this area, patient education of effective treatments should be improved among health care personnel.
